# Establishing a quantitative link between crystal violet absorbance and biomass in biofilms

**DOI:** 10.1016/j.mex.2025.103630

**Published:** 2025-09-16

**Authors:** Sheida Stephens, Radhakrishnan Mahadevan, D. Grant Allen

**Affiliations:** a Department of Chemical Engineering and Applied Chemistry, University of Toronto, 200 College St., Toronto, Ontario M5S 3E5, Canada; b Institute of Biomaterials and Biomedical Engineering, University of Toronto, Toronto, Ontario, Canada

**Keywords:** Bioprocess, Biofilms, Crystal violet assay, Purple bacteria, Optimization, Collaboration

## Abstract

Quantifying biomass is essential for studying biofilms in biomanufacturing, yet widely used assays such as crystal violet (CV) often yield results that are difficult to compare across laboratories due to its reliance on absorbance as a subjective proxy for biomass. To address this, we present a standardized method that calibrates CV absorbance against cellular optical density (OD) and dry cell weight (DCW) using centrifuged planktonic cultures. By establishing a three-way correlation among OD, DCW, and CV absorbance, this simple method allows for quantitative, reproducible, and comparable biomass measurements. Validation across *Escherichia coli* strains and *Rhodopseudomonas palustris* showed strong linearity, particularly when using 10% acetic acid as the solvent. Seasonal and instrument-based variability were evaluated, supporting the method’s robustness and broad utility. This method enables researchers to normalize CV data, improving inter-laboratory comparability and supporting more accurate assessment of biofilm productivity.

## Graphical abstract

**Figure:**
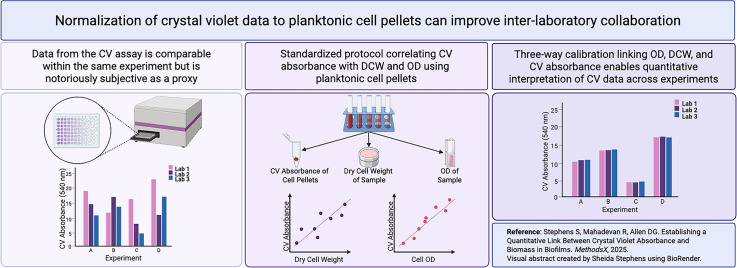

Key features of the method:

• Converts CV absorbance into a quantitative, biomass-normalized metric
• Enables reproducible biomass measurement across strains and conditions
• Supports cross-laboratory comparison through standardization

Specifications table

**Table:** 

Subject area	Engineering
More specific subject area	Biofilm quantification
Name of your method	Planktonic cell pellet crystal violet assay
Name and reference of original method	G.D. Christensen, W.A. Simpson, J.J. Younger, L.M. Baddour, F.F. Barrett, D.M. Melton, E.H. Beachey, Adherence of coagulase-negative staphylococci to plastic tissue culture plates: A quantitative model for the adherence of staphylococci to medical devices, J Clin Microbiol 22 (1985) 996–1006. https://doi.org/10.1128/jcm.22.6.996–1006.1985.

## Background

In nature, biofilms represent one of the most prevalent modes of microbial life [1]. Established through the secretion of extracellular polymeric substances (EPS) that facilitate adhesion to surfaces and to neighboring cells [2,3], this matrix forms a cohesive film composed of proteins, glycoproteins, glycolipids, and extracellular DNA, enabling bacteria to develop structured, cooperative communities [4,5]. Biofilm formation provides a protective environment that shields cells from nutrient deprivation, pH fluctuations, oxidative stress, biocides, and antimicrobial agents [6,7]. Within a biofilm, bacterial cells function as a coordinated community, exhibiting complex behaviors—including the development of channel-like structures that serve roles similar to a circulatory system or a coral reef [5,6,8].

Despite their biological advantages, biofilms can pose significant challenges in applied contexts. In industrial systems, they contribute to pipeline corrosion and flow obstruction [2,9]. In medical settings, biofilms are associated with persistent infections and increased antibiotic resistance [10,11]. However, in recent years, applications have been developed for which one can harness this growth mode including in wastewater treatment [12], bioremediation [13], and hydrogen production [14]. These applications capitalize on the capacity of biofilms to support high cell densities and establish microenvironments that facilitate nutrient exchange and waste removal.

**Fig. 1:**
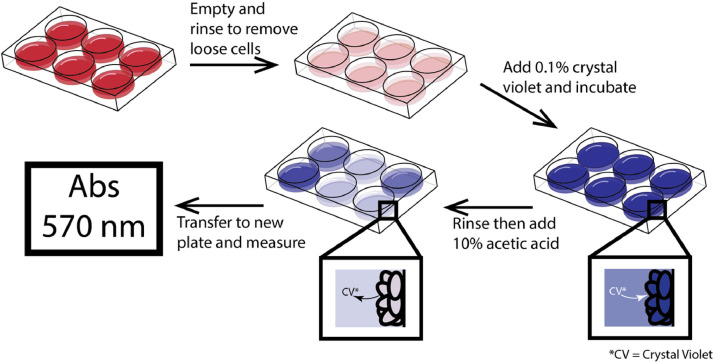
Microtiter plate assay procedure. This illustration is simplified from 96 wells to 6 wells. Once the biofilm growth experiment is complete in a microtiter plate, the plate must be emptied and rinsed to remove all planktonic cells then 0.1 % crystal violet is added to the wells and left to incubate to allow cells to take up the dye. Then plates must be rinsed to ensure no loose dye remains and add a solvent like 95 % ethanol or 10 % acetic acid to pull the dye from the cells to allow for cellular quantification by absorbance measurement.

Regardless of the application, standardized quantification of biofilms remains limited. Both direct and indirect methods are available. Direct methods quantify cell number through approaches such as microscopic counts or Coulter counting. Indirect methods rely on proxies like total carbon content or ATP bioluminescence. These methods have been comprehensively reviewed by Wilson et al. [15] and will not be discussed in detail here. Among the most commonly used quantification techniques is the crystal violet (CV) microtiter plate assay, originally described by Christensen et al. [16] as illustrated in Fig. 1. This method involves staining biofilms with crystal violet dye, eluting the dye with a solvent (e.g., 95 % ethanol or 10 % acetic acid), and measuring absorbance via spectrophotometry. Higher absorbance values correspond to greater biofilm mass.

This method offers multiple advantages: it is simple, cost-effective, and amenable to high-throughput screening since each well in a microtiter plate can represent a unique condition [15,17,18]. Furthermore, it is adaptable to alternative biofilm growth formats, such as culture tubes or surface coupons. Nonetheless, a key limitation of the method is its comparative nature. Absorbance values are interpreted relative to other wells in the same plate or tubes within the same experiment and are not typically correlated to an objective measure such as dry cell weight. While the assay may be reproducible within a given lab, results are influenced by variables such as lab consumables used, surface properties, user technique, and equipment calibration [17,18]. Additionally, methodological adaptations can further compromise inter-laboratory comparability.

**Fig. 2:**
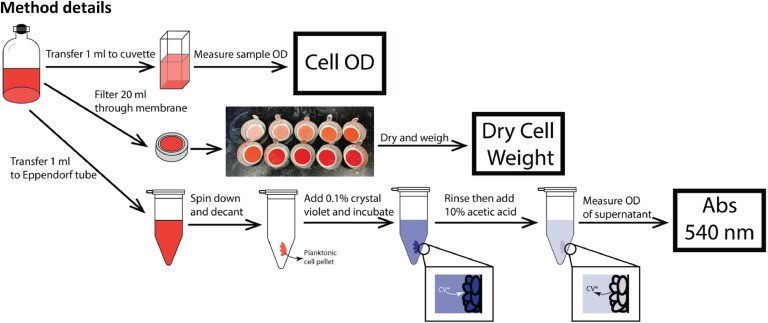
Schematic of the proposed protocol for correlating crystal violet absorbance with dry cell mass and OD. Samples are divided into aliquots for OD measurement (1 mL), dry weight determination (20 mL filtered), and crystal violet staining (1 mL pelleted).

To address these limitations, we propose a standardized protocol (Fig. 2) that correlates CV absorbance with dry cell weight using planktonic cell pellets. Our objective is to introduce a normalization step that enables CV data to be converted into a DCW measurement that can be interpreted quantitatively and compared across laboratories.

One of the first calibration steps when working with a new bacterial strain is establishing the relationship between OD and DCW, enabling biomass estimation from OD—a subjective proxy. Here, we propose extending this calibration by introducing a third parameter: crystal violet absorbance. Establishing this three-way correlation between OD, DCW, and crystal violet absorbance provides a framework for standardizing the widely used crystal violet assay and improving reproducibility across researchers and laboratories.

## Method details

### Instruments and equipment

- Conical bottom Eppendorf tubes
- 96 well microtiter plate
- 0.2 µm filter paper or membrane
- Microcentrifuge
- Spectrophotometer
- Plate reader
- Micropipettes and tips
- Beakers
- Deionized pure water
- 0.1 % (w/v) crystal violet solution
- 95 % ethanol or 10 % acetic acid
- Analytical balance
- Container for liquid waste

### Procedure

1. Cultivate bacterial cells in suspended culture according to strain-specific protocols.
2. Prepare a dilution series (minimum 23 mL per sample) covering a range of OD values (e.g., 0.1 to 2.0).
3. Quantify planktonic cells:

a. Measure 1 mL of each dilution in a spectrophotometer to confirm OD.
b. Measure 100 µL of each dilution in a plate reader (e.g., OD600 for *Escherichia coli*).

4. Determine dry cell weight (DCW):

a. Pre-weigh filter paper or membrane.
b. Filter 20 mL of each sample through the membrane and dry to constant weight (air-dried or in oven).
c. *Re*-weigh and subtract initial mass to determine DCW.

5. Measure crystal violet absorbance:

a. Transfer 1 mL of each dilution to an Eppendorf tube.
b. Centrifuge at 10,000 G to pellet cells.
c. Decant supernatant and wash cells with deionized water.
d. Repeat centrifugation and decant.
e. Add 250 µL of 0.1 % crystal violet and resuspend.
f. Centrifuge and decant excess stain.
g. Gently wash the pellet by submerging the pellet in a beaker of clean deionized water (being careful not to spill into clean water) and decant into waste container repeating until rinse water runs clean.
h. Add 375 µL of solvent (ethanol or acetic acid) to each tube and resuspend.
i. Centrifuge and transfer 100 µL of supernatant to a microtiter plate.
j. Repeat for all samples.
k. Include wells for blanks.
l. Measure absorbance at 540 nm using a plate reader.

6. Analyze data by plotting (ensure plate reader data is normalized to blanks):

a. Plate reader OD vs. spectrophotometer OD
b. Spectrophotometer OD vs. DCW
c. Crystal violet OD vs. DCW
d. Crystal violet OD vs. spectrophotometer OD

## Method validation

### Linearity

**Fig. 3:**
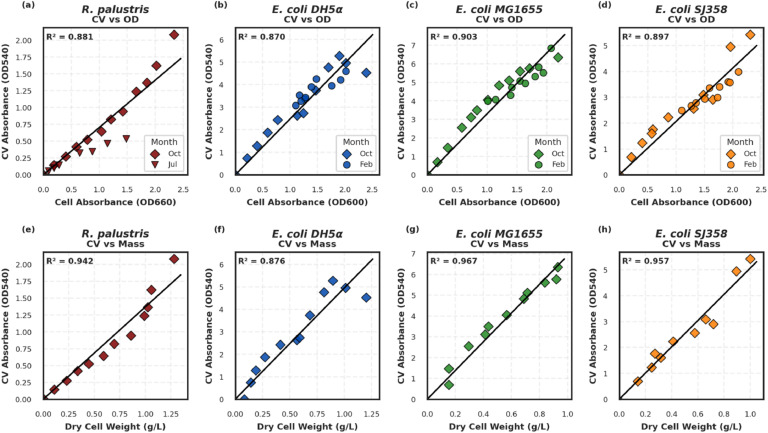
Relationship of crystal violet absorbance (OD540) using 10 % acetic acid with four different bacterial strains. Relationship against cell absorbance for a) R. palustris CGA009 at OD=660 nm. b) *E. coli* DH5α at OD=600 nm. c) *E. coli* MG1655 at OD=600 nm. d) *E. coli* SJ358 at OD=600 nm. Relationship against dry cell weight (g/L) for e) R. palustris CGA009 f) *E. coli* DH5α g) *E. coli* MG1655 h) *E. coli* SJ358. Linear regression was performed using ordinary least squares and the coefficient of determination (R^2^) of the aggregated data in each plot is shown.

To evaluate the linearity of the proposed method, experiments were conducted using *Rhodopseudomonas palustris CGA009* (Fig. 3a). A dilution series of suspended cultures was processed following the protocol described above. A strong correlation was observed between CV absorbance (measured at 540 nm) and optical density (OD660), indicating that CV staining reliably reflects cell concentration. Similar linear relationships were confirmed in three strains of *E. coli*—DH5α, MG1655, and SJ358 (Figs. 3b, 3c, 3d, respectively). When absorbance was plotted against dry cell weight (Figs. 3e–3h), linearity persisted, further validating the robustness of the approach.

The gentle washing procedure detailed in this protocol, which eliminates pipetting during decanting, was effective in minimizing cell loss. This is evidenced by data collected in July and October (Fig. 3a), where the use of gentle decanting in October resulted in markedly reduced variability and improved linearity compared to the July data, in which pipetting was used. These results support previous findings that vigorous pipetting may contribute to inconsistencies in biofilm staining protocols [18].

### Solvent comparison

**Fig. 4:**
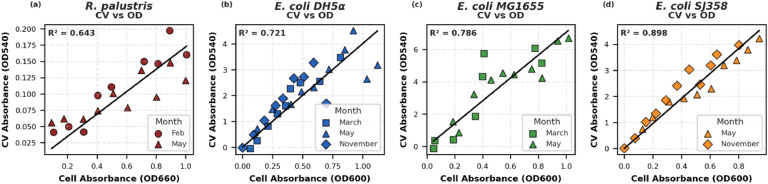
Relationship of crystal violet absorbance (OD540) using 95 % ethanol with four different bacterial strains. Relationship against cell absorbance for a) R. palustris CGA009 at OD=660 nm. b) *E. coli* DH5α at OD=600 nm. c) *E. coli* MG1655 at OD=600 nm. d) *E. coli* SJ358 at OD=600 nm. Linear regression was performed using ordinary least squares and the coefficient of determination (R^2^) of the aggregated data in each plot is shown.

As previously stated, different solvents can be used to extract CV from the cells for quantification. We have shown the data for 10 % acetic acid (Fig. 3), but 95 % ethanol is also a common and effective solvent for CV extraction. As shown in Fig. 4, ethanol enables linear quantification in all tested strains, with especially high correlation observed for *E. coli* SJ358 (Fig. 4d). However, data collected using ethanol exhibited increased variability compared to acetic acid. This may be attributed to ethanol's low surface tension and high volatility, which complicate pipetting and volume control. Additionally, ethanol may be less effective at solubilizing crystal violet, as previously reported [19].

### Repeatability

To demonstrate repeatability, the full protocol was executed at multiple time points across one year, encompassing different seasons and laboratory conditions. For *R. palustris*, data using acetic acid were collected in July and October (Fig. 3a) and using ethanol in February and May (Fig. 4a), with consistently strong linear trends. Similar consistency was observed across all *E. coli* strains. For example, *E. coli* DH5α was tested with acetic acid in October and February (Fig. 3b) and with ethanol in November, March, and May (Fig. 4b). Repeat experiments with MG1655 (Figs. 3c and 4c) and SJ358 (Figs. 3d and 4d) further reinforce the method’s reproducibility.

### Biofilm relevance

The protocol developed in this study uses centrifuged planktonic cells along the inner walls of Eppendorf tubes. These structures mimic the accumulation observed in true biofilms but do not include the full matrix of EPS. Since CV binds non-specifically to negatively charged molecules, this assay measures both cellular and matrix material [20]. Consequently, measurements from true biofilms may differ from planktonic cell pellets.

To explore this, we compared CV absorbance in cell pellets versus colony-derived biofilms of *E. coli* DH5α, MG1655, and SJ358. We initiated biofilm growth using the colony biofilm assay [[21], [22], [23]]: a common method that involves vacuum filtering cells through sterile 0.2 µm polycarbonate membranes and letting them grow on LB agar plates. We let each plate grow for 2 days in a 37 °C static incubator before resuspending the cells and matrix in varying amounts of deionized water to create a range of OD600 values. The solutions were then vacuum-filtered, dried, and the dry cell weight was measured.

**Fig. 5:**
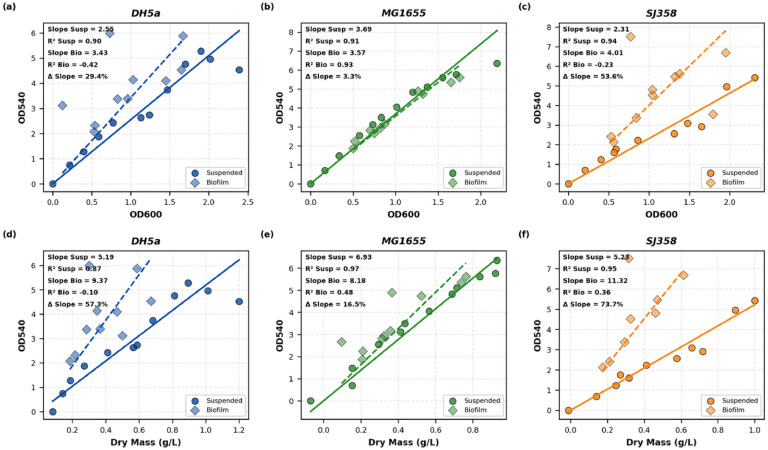
Comparing *E. coli* samples grown as a biofilm and resuspended in deionized water (biofilm data, diamonds) to planktonic cell pellet protocol (suspended data, circles). The relationship between crystal violet absorption (OD540) and cell absorption (OD600) is shown using three different strains of *E. coli* (a) DH5α (b) MG1655 and (c) SJ358. The relationship between crystal violet absorption (OD540) and dry cell weight is shown using *E. coli* (d) DH5α (e) MG1655 and (f) SJ358. Linear regression was performed using ordinary least squares and the coefficient of determination (R^2^) of the data is shown. The slope of each line was then determined and the percent difference between the slopes was calculated.

As shown in Fig. 5, biofilms formed by DH5α (Fig. 5a, d) and SJ358 (Fig. 5c, f) are underestimated by the planktonic cell pellet method, whereas those formed by MG1655 (Fig. 5b, e) are well-approximated. This indicates that, in certain strains, CV absorbance is higher in biofilms than in an equivalent number of suspended cells. This discrepancy arises from the nonspecific binding of CV and the strain-dependent variation in EPS composition [20]. As a result, two strains with comparable cell counts may yield substantially different CV absorbance values [18,24]. Accordingly, while this method may provide a reliable estimate of cell mass for some strains, it may not be suitable for others. Preliminary experiments are therefore recommended to assess whether this approach is appropriate for direct quantification of total biofilm biomass in a given system.

### Instrument variability

**Fig. 6:**
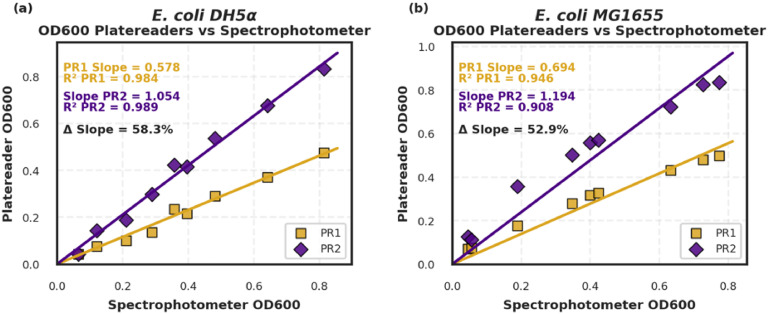
Comparing the data from two separate plate readers. Platereader 1 (PR1) and Platereader 2 (PR2) are two platereaders located in different laboratories at the University of Toronto. The samples were first measured by cuvette in a spectrophotometer and then a plate of all the samples was prepared and measured by the two platereaders. Samples were all taken minutes apart. Linear regression was performed using ordinary least squares and the coefficient of determination (R^2^) of the data is shown. The slope of each line was then determined and the percent difference between the slopes was calculated.

To highlight the importance of normalization, we measured OD600 of *E. coli* DH5α and MG1655 using three different instruments: a cuvette-based spectrophotometer and two microplate readers in different laboratories. All measurements were conducted within minutes of each other. As illustrated in Fig. 6, absorbance readings varied substantially between plate readers, with over 50 % difference in slope observed. This underscores the potential discrepancies that can arise when proxy metrics are used without calibration and reinforces the value of normalization to a physical parameter such as dry weight.

## Limitations

The original aim of this study was to develop a method of biofilm quantification that did not require the separation of a biofilm from its growth substrate through vortexing or scraping as most conventional methods require [15]. The theory was, if the baseline crystal violet absorption of the substrate was determined prior to any experimentation, we could determine an estimate for the cell count in the biofilm formation on said substrate at the endpoint of the experiment or by having numerous cultures running concurrently for sacrificial sampling on a time scale. However, our findings indicate that the accuracy of such estimates is highly dependent on the bacterial strain, owing to significant variability in the composition of EPS. The presence of various polysaccharides, for example, can give higher CV readings for a similar cell count [24]. As such, the direct application of this method for total biofilm biomass quantification is feasible only when validated for the specific organism in use.

Despite this limitation for this specific use, this method provides a robust framework for normalization. Establishing a relationship between crystal violet absorbance and dry cell weight enhances the reproducibility of crystal violet-based assays and facilitates more consistent communication among researchers. Therefore, standardizing this quantification step will not only improve intra-laboratory data coherence but also enable meaningful inter-laboratory comparisons and collaborations. We, thus, advocate for routine inclusion of this normalization step in biofilm research protocols. This standardized approach enables more accurate estimation of biofilm-associated biomass and can support consistent evaluation of bioproduction metrics, ultimately improving the comparability of research findings across studies.

## Related research article

None.

## Ethics statements

None.

## Supplementary material *and/or* additional information [OPTIONAL]

None.

## Declaration of competing interest

The authors declare that they have no known competing financial interests or personal relationships that could have appeared to influence the work reported in this paper.

## Data Availability

Data will be made available on request.
